# A Review on Transcriptional Responses of Interactions between Insect Vectors and Plant Viruses

**DOI:** 10.3390/cells11040693

**Published:** 2022-02-16

**Authors:** Michael A. Catto, Habibu Mugerwa, Brendon K. Myers, Sudeep Pandey, Bhabesh Dutta, Rajagopalbabu Srinivasan

**Affiliations:** 1 Department of Entomology, University of Georgia, Athens, GA 30602, USA; mac65630@uga.edu; 2 Department of Entomology, University of Georgia, Griffin, GA 30223, USA; hm00192@uga.edu (H.M.); sp36142@uga.edu (S.P.); 3 Department of Plant Pathology, University of Georgia, Tifton, GA 31793, USA; bkm23644@uga.edu (B.K.M.); bhabesh@uga.edu (B.D.)

**Keywords:** virus transmission, transmission modes, differential gene expression

## Abstract

This review provides a synopsis of transcriptional responses pertaining to interactions between plant viruses and the insect vectors that transmit them in diverse modes. In the process, it attempts to catalog differential gene expression pertinent to virus–vector interactions in vectors such as virus reception, virus cell entry, virus tissue tropism, virus multiplication, and vector immune responses. Whiteflies, leafhoppers, planthoppers, and thrips are the main insect groups reviewed, along with aphids and leaf beetles. Much of the focus on gene expression pertinent to vector–virus interactions has centered around whole-body RNA extraction, whereas data on virus-induced tissue-specific gene expression in vectors is limited. This review compares transcriptional responses in different insect groups following the acquisition of non-persistent, semi-persistent, and persistent (non-propagative and propagative) plant viruses and identifies parallels and divergences in gene expression patterns. Understanding virus-induced changes in vectors at a transcriptional level can aid in the identification of candidate genes for targeting with RNAi and/or CRISPR editing in insect vectors for management approaches.

## 1. Introduction

Plant viruses cause devastating yield losses in agricultural systems, which result in reduced economic productivity and nutritional insecurity globally [1]. Plant pathogenic viruses are transmitted mechanically, via seeds, and by vectors [2,3,4,5]. Vector-borne viruses are transmitted by insects, mites, nematodes, and fungi [6,7]. Several genera, species, or biotypes from major insect orders have specialized relationships with a wide range of viruses [8]. These relationships have varying degrees of complexity, which is dependent upon the mode of transmission. Ultimately, the specialized dynamics between plant pathogenic viruses and their associated vectors are critical for the successful spread of plant viruses.

Several factors have been attributed to the emergence and successful establishment of plant viruses in a wide array of agricultural systems. These include: extensive monoculture of crops that are susceptible to both viruses and vectors, the movement of virus-infected plant materials and insect vectors from their native to new environments, plant viruses’ ability to rapidly evolve and adapt, and the effect of climate change on the distribution area of hosts and vectors [9,10,11,12,13,14,15,16]. Many plant viruses also possess a broad host range, which in turn aids in their successful establishment in new environments [17].

Among the various insect orders known to transmit plant viruses, insects with piercing and sucking mouthparts comprise the majority [18]. These include aphids (Hemiptera, Aphididea), whiteflies (Hemiptera, Aleyrodidae), leafhoppers (Hemiptera, Cicadellidae), and thrips (Thysanoptera, Thripidae) [19,20]. The geographical distribution of aphids tends to be predominantly in temperate regions, and aphids are major pests of crops such as cereals including wheat and barley [21,22]. Whiteflies are known to be prevalent in sub-tropical and tropical regions and certain members in the cryptic species complex are pests on a variety of vegetable and row crops such as cotton [23,24]. Leaf and planthoppers are pests of host plants such as potato, rice, and soybean in temperate, sub-tropical, and tropical regions of the world [25,26]. Thrips are pervasive in sub-tropical and tropical regions throughout the world and are known pests for a variety of vegetable and row crops, such as tomato and peanut, respectively [27,28].

Insects belonging to the order Hemiptera and Thysanoptera transmit plant viruses in 12 (Begomovirus, Carlavirus, Closterovirus, Crinivirus, Cucumovirus, Ipomovirus, Mastrevirus, Potyvirus, Polerovirus, Phytoreovirus, Tenuivirus, and Torradovirus) and 5 (Orthotospovirus, Ilarvirus, Carmovirus, Sobemovirus, and Machlomovirus) genera, respectively [29,30,31,32]. Additionally, beetles (order Coleoptera) are also reported to transmit over 40 different plant viruses [33]. Details on the specific vector and virus taxonomic classifications from studies discussed in this review are listed in Table 1. Some viruses in the above-listed genera have been ranked in the top ten most agriculturally important plant viruses [34,35]. Plant viruses in the above-mentioned genera diversely interact with their insect vectors, and these interactions influence virus persistence within the insect [36,37,38,39,40].

**Figure 1:**
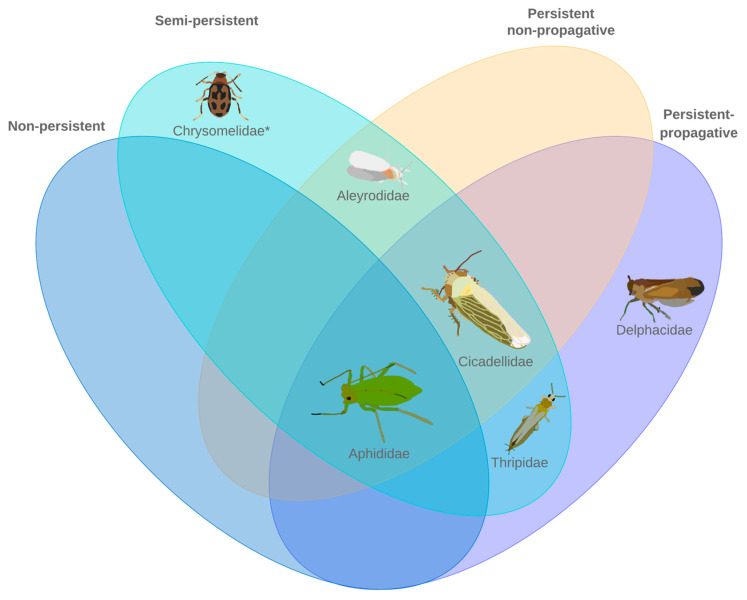
The six most common plant virus transmitting insect families representing three insect orders. Five of the six families have been investigated for differential gene expression in relation to phytovirus infection. * No differential gene expression studies have been conducted as of December 2021.

The interactions between a given vector and the associated virus could differentially impact gene expression in the vectors (Figure 1). To investigate the differential expression patterns in detail, various tools and pipelines have been developed and are detailed in this review. A de novo transcriptome assembly can provide a foundation for determining differentially expressed genes (DEGs) [41]. To attain greater clarity as to the genetic landscape, access to a high-quality genome assembly would be essential. In the case that a reference genome is available, a de novo transcriptome assembly may still be necessary to account for potentially misassembled genes or improper annotations [42,43]. Whole genome assemblies are available for most insect vectors discussed in this review (Table 1). For non-model organisms, a de novo transcriptome or genome assembly is required prior to differential expression analysis.

**Table 1:** Taxonomy of vector–virus associations in which transcriptional responses pertinent to different modes of virus transmission were evaluated. Virus species descriptions are based on the March 2021 International Committee on Taxonomy of Viruses (ICTV) report [44].

Insect Family	Insect Binomial Name	Insect Common Name	Virus Family	Virus Genus	Virus Species	Reference
Non-persistent						
Aphididae	*Myzus persicae*	Green peach aphid	*Bromoviridae*	*Cucumovirus*	*Cucumber mosaic virus*	[45]
Semi-persistent						
Aleyrodidae	*Bemisia tabaci* Middle East-Asia Minor 1 (MEAM1)	Sweetpotato whitefly	*Closteroviridae*	*Crinivirus*	*Tomato chlorosis virus*	[46]
					*Cucurbit yellow stunting disorder virus*	[47]
	*Bemisia tabaci* Mediterranean (MED)				*Tomato chlorosis virus*	[48]
Cicadellidae	*Graminella nigrifrons* *	Black-faced leafhopper	*Secoviridae*	*Waikavirus*	*Maize chlorotic dwarf virus*	[49]
Persistent non-propagative						
Aleyrodidae	*Bemisia tabaci* (MEAM1)	Sweetpotato whitefly	*Geminiviridae*	*Begomovirus*	*Tomato yellow leaf curl China virus*	[50,51,52,53]
					*Tomato yellow leaf curl virus*	[54,55,56]
	*Bemisia tabaci* (MED)					[48]
Aphididae	*Schizaphis graminum*	Greenbug	*Solemoviridae*	*Polerovirus*	*Cereal yellow dwarf virus-RPV*	[57]
	*Acyrthosiphon pisum*	Pea aphid		*Enamovirus*	*Pea enation mosaic virus 1*	[58]
	*Sitobion avenae*	English grain aphid		Unassigned species	*Barley yellow dwarf virus GAV*	[59]
Persistent-propagative						
Thripidae	*Frankliniella occidentalis*	Western flower thrips	*Tospoviridae*	*Orthotospovirus*	*Tomato spotted wilt orthotospovirus*	[60,61,62,63,64]
	*Frankliniella fusca* *	Tobacco thrips				[65]
	*Thrips palmi* *	Melon thrips			*Capsicum chlorosis orthotospovirus*	[66]
Delphacidae	*Nilaparvata lugens*	Brown planthopper	*Rhabdoviridae*	*Alphanucleorhabdovirus*	*Maize mosaic alphanucleorhabdovirus*	[67]
	*Laodelphax striatellus*	Small brown planthopper	*Phenuiviridae*	*Tenuivirus*	*Rice stripe tenuivirus*	[68,69,70]
			*Reoviridae*	*Fijivirus*	*Southern rice black-streaked dwarf virus*	[71]
	*Sogatella furcifera*	Whitebacked planthopper				[72]
	*Peregrinus maidis*	Corn planthopper	*Rhabdoviridae*	*Alphanucleorhabdovirus*	*Maize mosaic alphanucleorhabdovirus*	[73]
Cicadellidae	*Graminella nigrifrons* *	Blackfaced leafhopper		*Gammanucleorhabdovirus*	*Maize fine streak gammanucleorhabdovirus*	[74,75]
			*Secoviridae*	*Waikavirus*	*Maize chlorotic dwarf virus*	[49]

* Organisms for which no genome was available as of December 2021.

## 2. Profiling Transcriptional Responses

### 2.1. Genetic Foundations

RNA sequencing and bioinformatics analyses are powerful techniques for unraveling the complex interactions between viruses and their vectors. A comparison of transcriptomes between non-viruliferous and viruliferous vectors can aid in understanding qualitative and quantitative differential gene expression modulated by the virus. RNA-Seq methods offer a valuable strategy for sequencing transcripts in rapid, accurate, and informative ways. Studies focused on the six insect families noted in this review have been steadily increasing over the past 10 years (Figure S1). Most of the studies conducted on transcriptomic datasets are based on whole insects rather than specific tissues, mainly due to the limitations of isolating various tissue types [76,77,78] (Figure S2).

**Figure 2:**
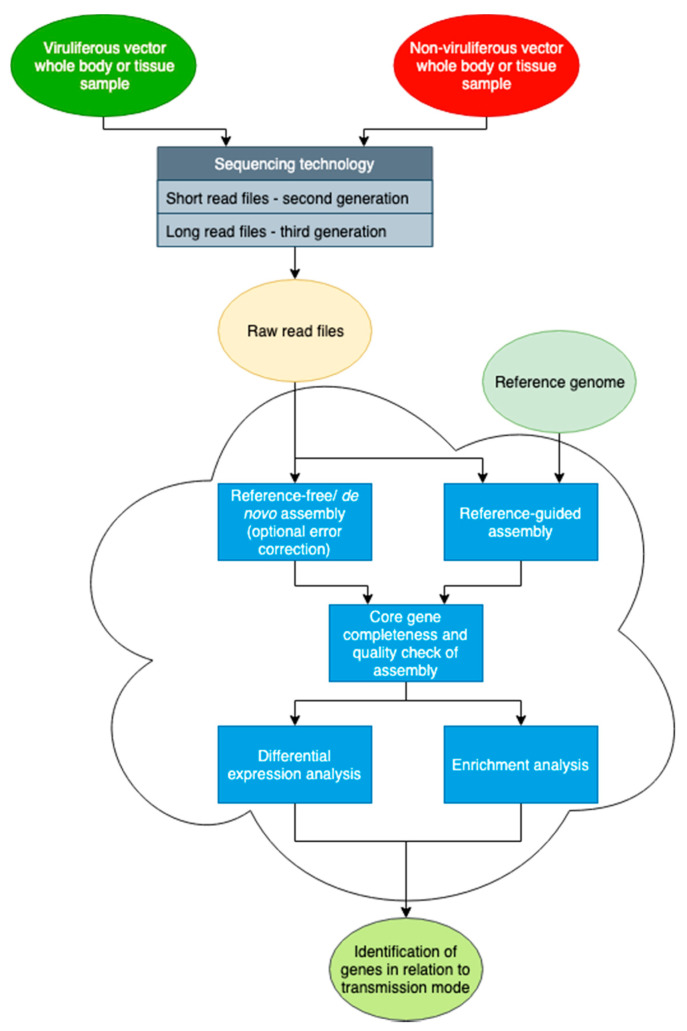
Diagram detailing a potential pipeline to derive an assembled transcriptome. Initial steps involve the generation of raw data sets from sequencing facilities. Error correction of long read sequences with short reads may be necessary in some cases. The assembly of a transcriptome may be completed de novo if a genome and gene models are not available. The differential expression profile is then determined by mapping the raw read sequences to the assembled genome. Additionally, Gene Ontology term enrichment helps make logical associations between the differentially expressed genes and a given phenotype. See Supplementary File for references.

For experiments that seek to answer questions about gene regulation, the easiest way to represent how well up or downregulated a gene is to determine the differential expression profile of a viruliferous group against a non-viruliferous group of organisms. In most cases, if not all, this produces an accurate example of the expression levels. The Gene Ontology (GO) database or the Kyoto Encyclopedia of Genes and Genomes (KEGG) database can assign functions to these proteins [79,80]. Having a reference genome provides high levels of certainty as to whether a given gene of interest is up or downregulated [81]. A basic roadmap for the assembly and differential expression analysis is included in Figure 2.

To attain a concrete resolution on the gene expression profile in each insect/vector, an assembled genome is often an invaluable asset. As sequencing costs are decreasing, more insect genomes are being assembled and projects such as i5k initiative are enabling this knowledge base to grow [82,83]. Some insects, such as the sweetpotato whitefly Middle East-Asia Minor 1 (MEAM1), *Bemisia tabaci* (Gennadius), have a genome database with a highly curated draft genome, while there is not always a database for other insect vectors [84]. Several aphid genomes have been assembled and annotated, with the first one being the pea aphid, *Acyrthosiphon pisum* (Harris), and its genome database is known as the AphidBase [85,86]. Additionally, two more aphid genomes have been annotated: the soybean aphid, *Aphis glycines* (Matsumura), and the wooly apple aphid, *Eriosoma lanigerum* (Hausmann) [87,88]. Three planthopper genomes have been fully assembled as well; the first was the brown planthopper, *Nilaparvata lugens* (Stål), followed by the whitebacked planthopper, *Sogatella furcifera* (Hovárth), and the small brown planthopper, *Laodelphax striatellus* (Fallén) [89,90,91]. Recently, all three planthoppers’ genomes were revisited with higher resolution from PacBio sequencing [92]. In Thysanoptera, the genome of the western flower thrips, *Frankliniella occidentalis* (Pergande), was assembled and annotated [93].

### 2.2. Transmission Modes

The successful transmission of plant viruses is dependent on the nature of the interactions between plant viruses, vectors, and host plants [38,39,40,94,95,96]. Through modulation of plant phenotype by altering plant physiology, such as coloration, nutrients, and volatile organic compounds, plant viruses can influence insect vector behavior and fitness, thereby favoring their transmission [97,98,99]. Currently, there are four described major modes of virus transmission, ranging from non-persistent to persistent-propagative [36,37]. Irrespective of the mode of transmission, many plant viruses infecting their host plants tend to attract their associated vectors [97,100,101]. However, the arrestment duration of their vectors ranging from a brief period (without providing long-term fitness benefits) to a prolonged period (with provision of long-term fitness benefits) is dependent on the mode of transmission [97]. On one end of the spectrum, non-persistent viruses (NPVs), such as cucumber mosaic virus (CMV), briefly arrest their aphid vectors, thereby facilitating rapid virus acquisition and vector dispersal, presumably aided by poor host plant quality [98]. On the opposite end of the spectrum, persistent-propagative viruses, such as tomato spotted wilt orthotospovirus (TSWV), arrest the thrips vectors for longer periods to enable virus acquisition and inoculation thereafter [102,103]. The prolonged feeding by vectors of persistently transmitted viruses is linked to improved plant quality in virus-infected host plants [97,102]. These diverse macro-level effects of plant viruses on their vectors have led researchers to investigate plant viruses-modulated micro-level effects on their vectors using transcriptomic and genomic tools (Table 2).

**Figure 3:**
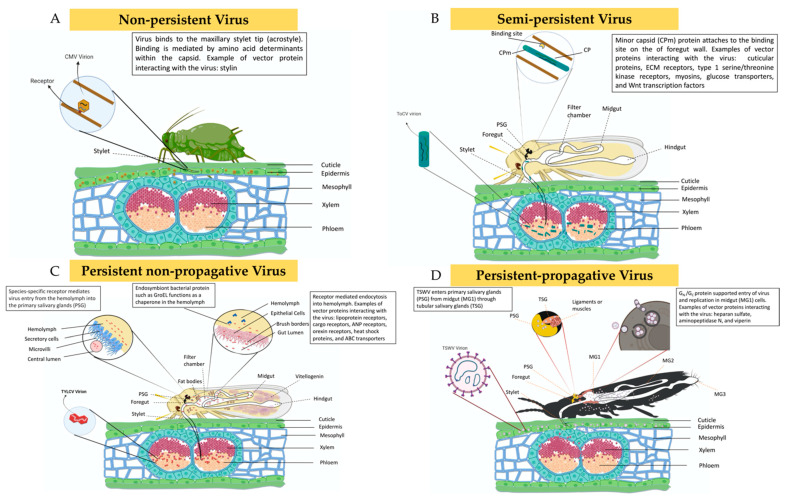
Schematic diagram explaining the interactions between plant viruses and their vectors with respect to different transmission modes viz., non-persistent, semi-persistent, persistent non-propagative, and persistent-propagative. Modeled after [29,94,95,96,107]. (**A**) Non-persistent viruses, such as cucumber mosaic virus (CMV), are acquired by aphids from the epidermal cells of infected plants and retained at the tip of its stylet (acrostyle) at the distal end of the common (food/salivary) duct. (**B**) Semi-persistent viruses, such as tomato chlorosis virus (ToCV), are phloem-limited in infected plants, and the virus attaches to the binding site at the vector’s foregut with the help of the minor capsid protein (CPm). (**C**) Persistent non-propagative viruses, such as tomato yellow leaf curl virus (TYLCV), are also phloem-limited and are retained in the midgut upon acquisition. Through receptor-mediated endocytosis, the virus traverses the midgut barrier into hemolymph where the endosymbiont protein GroEL interacts with the virion. The virus from the hemolymph reaches primary salivary glands mediated again via species-specific receptors. (**D**) Thrips acquire persistent propagative viruses, such as tomato spotted wilt virus (TSWV), from epidermal cells of infected plants. Gn/Gc protein supports virus entry into midgut cells, where replication of the virus occurs. The virus TSWV enters primary salivary glands (PSG) from MG1 through tubular salivary glands (TSG).

**Figure 4:**
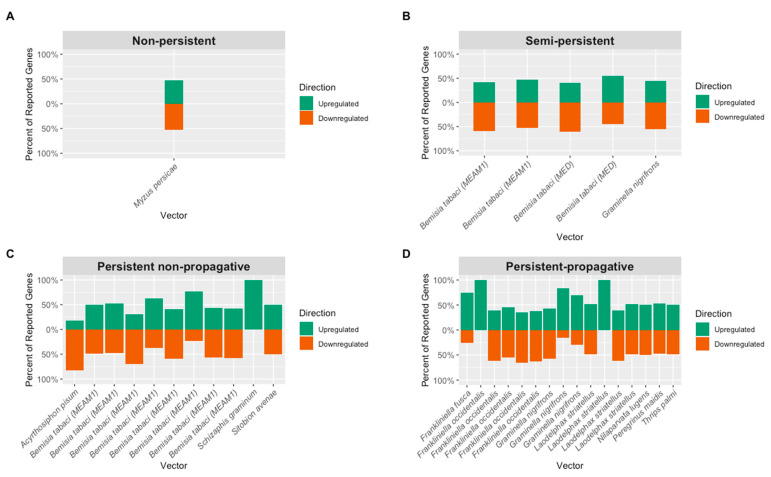
Comparison of upregulated and downregulated genes reported from studies focusing on (**A**) non-persistent, (**B**) semi-persistent, (**C**) persistent non-propagative, and (**D**) persistent-propagative transmission. Note that studies with 100% upregulation had very few genes reported due to the method chosen.

Non-persistent viruses have short acquisition periods and inoculation periods, lasting only a few seconds, with no latent period [29,104,105,106,107,108,109]. There are currently two documented strategies NPVs use to interact with the receptors of their vectors; these are the “capsid,” and the “helper component” strategies. In the “capsid” strategy, the coat protein (CP) of the virus interacts directly with the vector’s binding sites (Figure 3A) [29,108], while in the “helper component” strategy, the CP interacts indirectly with vector’s binding sites (cell-surface receptor) through the non-structural helper component protein [29]. Viruses that use this mode of transmission often are spread by aphids due to their swift probing behavior associated with assessing host plant suitability [29,104]. NPVs are non-tissue specific and can be transmitted by insects within a short feeding window [19]. NPVs arguably possess the least complicated strategy in terms of virus-vector interaction, followed by the semi-persistent viruses (SPVs). At the time of this review, only one study had examined the transcriptional responses of vector following the acquisition of a NPV (Figure 4A). Liang et al. focused on the impact of CMV on the gene expression profile of the green peach aphid, *Myzus persicae* (Sulzer) [45].

Despite the prolonged retention of SPVs by their vectors, these viruses share some similarities with NPVs. For example, SPVs may require helper components to bind to the vector’s foregut (Figure 3B) [29,104]. NPVs may interact with helper components from related viruses to facilitate their transmission, which also may occur with SPVs during co-inoculation [29]. The primary difference between the two transmission strategies is where viruses localize within the vector. Semi-persistently transmitted viruses such as maize chlorotic dwarf virus (MCDV) are phloem-limited in their hosts and typically localize within the foregut of their leafhopper vectors, as opposed to exclusively being stylet-borne as in the case of NPVs [110,111]. Under semi-persistently transmitted viruses, this review focuses on two whitefly-transmitted viruses (tomato chlorosis virus (ToCV) and cucurbit yellow stunting disorder virus (CYSDV)) and one leafhopper-transmitted virus, MCDV. At the micro-level, transcriptional responses relating to virus acquisition have been explored in two whitefly cryptic species, *B. tabaci* (MEAM1) and Mediterranean (MED), as well in the blackfaced leafhopper, *Graminella nigrifrons* (Forbes) (Figure 4B). Furthermore, beetles from the family Chrysomelidae can transmit plant viruses, such as the bean pod mottle virus (BPMV), semi-persistently [20,109,112]. However, there are no reported studies on the gene expression level changes induced by viruses in leaf beetles.

Persistent viruses enter their vector through the feeding organs, move through the midgut lumen, translocate across the walls of epithelial cells into the hemolymph, and ultimately travel to the salivary glands; from there, the virions are inoculated into non-infected plants while feeding [19]. This mode of transmission is categorized into non-propagative and propagative, with viruses in the former being incapable of replicating within the insect tissues; while in the latter, the viruses multiply within the host tissues, such as the midgut, salivary gland, and fat body (Figure 3C) [36]. Under the persistent non-propagative category, extensive work has focused on two whitefly-transmitted viruses: tomato yellow leaf curl virus (TYLCV) and tomato yellow leaf curl China virus (TYLCCNV). Additionally, some aphids, such as the greenbug, *Schizaphis graminum* (Rodani), and the English grain aphid, *Sitobion avenae* (Fabricius), are known to transmit cereal yellow dwarf virus-RPV (CYDV-RPV) and barley yellow dwarf virus GAV (BYDV-GAV), respectively, in a persistent and non-propagative manner [57,59]. Most studies in this category have focused on the transcriptional responses in *B. tabaci* (MEAM1) (Figure 4C).

In the persistent-propagative category, extensive work has focused on transcriptional responses in thrips, leafhoppers, and planthoppers (Figure 4D). Persistent-propagative viruses are thought to have originated from animal-infecting viruses, of which several are still capable of infecting insects and are also reported to be transmitted vertically to the next generation [6,111]. Listed below are various pathosystems of persistent-propagative viruses highlighted in this review. In thrips, the virus is acquired during their first instar stage, and it is retained throughout the pupal and adult molts (Figure 3D) [113]. TSWV is one of the most important viruses transmitted by nine thrips species, and gene expression studies have mainly focused on *F. occidentalis* and tobacco thrips, *Frankliniella fusca* (Hinds) [60,61,62,63,64,65,114]. Capsicum chlorosis virus (CaCV) is another important virus transmitted by the melon thrips, *Thrips palmi* (Karny), in Australia and Southeast Asia [115,116]. There are no transcriptional studies on CaCV-infected and non-infected melon thrips. The maize mosaic rhabdovirus (MMV) and maize stripe tenuivirus (MSTV) are viruses transmitted by the corn planthopper, *Peregrinus maidis* (Ashmead), in a persistent and propagative manner. The rice stripe virus (RSV) and Southern rice black-streaked dwarf virus (SRBSDV) are the other persistent and propagative viruses transmitted by *L. striatellus* and *S. furcifera*, respectively [19,117,118,119].

## 3. Plant-Virus-Induced Responses in Vectors

### 3.1. Cell-Surface Reception and Virus Tropism

Virion binding to cell-surface receptors facilitates cell entry. Numerous putative cell surface receptors have been identified via transcriptomics, and their diversity seems to vary with virus species and mode of transmission by vectors.

Cuticular proteins present at the stylet tip, such as the stylins of aphids, have been identified as putative receptors of stylet-borne non-persistent viruses [45,120]. Transcripts of aphid cuticular protein genes were associated with the cell entry of zucchini yellow mosaic virus (ZYMV) and with *M. persicae* and CMV [45,121]. Cuticular protein gene transcripts were also differentially expressed in vectors associated with the transmission of semi-persistent viruses. Five cuticular proteins’ encoding genes were upregulated in *B. tabaci* (MEAM1) that acquired CYSDV from infected melon, *Cucumis melo* (L.), plants [47]. In addition to cuticular proteins, extracellular matrix (ECM)-receptors, orphan genes, neuroactive-ligand receptors, and type 1 serine/threonine kinase receptor proteins were identified among the DEGs in *B. tabaci* (MEAM1) that acquired ToCV—another semi-persistent virus [46]. ECM receptors are transmembrane proteins that are reported as viral receptors favoring viral recognition, attachment, and entry into the cell [122]. Type 1 serine/threonine kinase receptors are ubiquitous transmembrane proteins involved in cell entry of viruses, such as rabies virus [123,124]. The ECM receptors were downregulated, while the neuroactive-ligand and type 1 serine/threonine kinase receptors were upregulated in whiteflies that acquired ToCV [46]. Twenty-one more orphan genes in *B. tabaci* (MEAM1) that acquired ToCV and CYSDV were downregulated than in non-viruliferous insects [47]. Some of the orphan genes in *B. tabaci* (MEAM1) that acquired CYSDV and ToCV were speculated to play a role in virus attachment to the vector’s foregut. Orphan genes were also reported in other arthropods to encode surface antigens that are involved in interactions between the viruses and their hosts [125].

In whiteflies that acquired the persistent virus, TYLCV, from infected tomato plants, low-density lipoprotein receptor (LDLR) was upregulated in *B. tabaci* (MEAM1) [54]. LDLRs are cell-surface proteins involved in the receptor-mediated endocytosis of viruses such as feline leukemia virus (FeLV), hepatitis C virus (HCV), and vesicular stomatitis virus (VSV) [126,127]. Furthermore, in the midgut of *B. tabaci* (MEAM1) that acquired TYLCV, cargo receptors and epidermal growth factor (EGF)-like repeats, such as laminin subunit alpha-1, LDLR and EGF-like domain 8, were upregulated [55]. Cargo receptors are cell-surface proteins that bind simultaneously to cargo molecules (soluble proteins) and efficiently recruit them to nascent vesicles [128]. Geng et al. speculated that the upregulation of cargo receptors in viruliferous *B. tabaci* (MEAM1) could facilitate TYLCV endocytosis and crossover from the midgut epithelial cells into the hemolymph [55]. In *B. tabaci* that acquired another persistent virus from TYLCCNV-infected tobacco (*Nicotiana tabacum* L.) plants, a putative receptor, heparan sulfate proteoglycan (HSPG), was identified among the upregulated expressed sequence tags [51]. Numerous viruses, such as the herpes simplex virus, were shown to bind to the HSPG receptor, thereby enhancing virus entry into the target cell [129].

In thrips (*F. fusca*), homologs of heparan sulfate were identified upon the acquisition of a persistent and propagative orthotospovirus (TSWV), but this putative receptor was not identified in the transcriptome of the non-vector flower thrips, *Frankliniella tritici* (Fitch) [114]. Furthermore, in *F. fusca*, a homolog of aminopeptidase N, a known virus receptor in aphids, was among the upregulated genes in larvae and adults that fed on TSWV-infected peanut (*Arachis hypogaea* L.) plants [65].

Transcriptomic studies have revealed the presence of several putative receptors across transmission modes, especially in the persistent virus category. A few receptors appeared to aid in multiple transmission modes. Furthermore, a single virus seems to possess multiple putative receptors within the vector. These receptors are likely present in multiple tissues within a vector. The relevance of receptors’ diversity in the transmission mechanics of most plant viruses remains to be functionally validated. Nevertheless, transcriptomic resources have been of immense importance in establishing and/or speculating virus–vector receptor connections.

Besides receptors, numerous other proteins pertaining to virus tropism within vectors have been identified via transcriptomics. As expected, these proteins seem to vary across vectors and transmission modes. Four myosin genes were upregulated in *B. tabaci* (MEAM1) following acquisition of the foregut-borne semi-persistent CYSDV [47]. Myosins are molecular motors and have been documented to aid in the intracellular movement of plant viruses, such as tobacco mosaic virus in *Nicotiana benthamiana* (Domin.) [130]. Their role in interactions with a tissue-specific (foregut borne) virus as its aphid vector remains to be characterized. In another example involving a semi-persistent virus, Ding et al. reported five downregulated facilitative glucose transporters in *B. tabaci* (MED) adults that acquired ToCV from infected tomato plants [48]. By contrast, in a different study, 10 glucose transporters were upregulated in *B. tabaci* (MEAM1) that acquired ToCV [46]. Sugar transporters are composed of glucose molecules and are known to transport propagative arthropod viruses, such as *Bombyx mori* nucleopolyhedrosisvirus (BmNPV), across cells [131]. The role of transporters associated with a non-propagative and exclusively foregut borne virus, such as ToCV, in whiteflies remains to be ascertained.

The atrial natriuretic peptide (ANP) receptors, orexin 2 receptor, and sugar and amino acid transporters were differentially expressed in *B. tabaci* (MEAM1) that acquired a persistent circulative virus, TYLCV [54]. ANP receptors consist of four distinct domains: ligand-binding domain, transmembrane region, protein kinase-like homology domain and guanylyl cyclase domain [132]. Orexin receptors are natriuretic peptides and affect several circulatory and nervous system functions in higher animals [133,134]. The upregulation of ANP and orexin receptors in *B. tabaci* (MEAM1) that acquired TYLCV from infected tomato plants was implicated in regulating hemolymph circulation favoring virus movement [54]. Up and downregulation of various transporters in *B. tabaci* (MEAM1) that acquired TYLCV was linked to facilitating begomovirus movement across the midgut into hemolymph and into primary salivary glands thereafter [48]. In addition, heat shock proteins (HSPs) were upregulated in *B. tabaci* (MED) following TYLCV acquisition [48]. HSPs are reported to play a role in cell entry, replication and particle assembly, and movement in several animal viruses [135]. HSPs also are molecular chaperones responsible for cellular defense mechanisms against deleterious effects such as protein misfolding, degradation, and insoluble aggregation [136]. HSP 70, upregulated upon TYLCV acquisition in *B. tabaci*, is also believed to function as a chaperone [137].

Furthermore, in reference to TYLCV, ATP-binding cassette (ABC) transporters and knottin genes were among the identified DEGs in the midguts of *B.* *tabaci* (MEAM1) that acquired TYLCV [55]. ABC transporters are membrane proteins with diverse biological functions, including the unidirectional translocation of compounds across cellular membrane [138,139]. Geng et al. speculated that the 16 upregulated and 17 downregulated ABC transporters in viruliferous *B. tabaci* (MEAM1) could help TYLCV cross the midgut epithelial cells into the hemolymph [55]. Knottins are antimicrobial peptides and have been implicated in the circulative movement of TYLCV in *B. tabaci* [140,141]. The upregulation of knottin genes in viruliferous *B. tabaci* (MEAM1) midguts was speculated to be involved in TYLCV and TYLCCNV circulative transmission [51,55,140]. Facilitated trehalose and unknown protein transporters were among the identified upregulated DEGs in *B. tabaci* (MEAM1) that acquired TYLCV [56]. These genes were speculated to play a crucial role in TYLCV transport in the hemolymph [54,55].

A few transport- and/or tropism-pertinent genes were associated with foregut borne semi-persistent viruses. By contrast, it is logical to identify several genes associated with these functions with persistent circulative viruses that interact with multiple tissue types within the vector.

### 3.2. Virus Replication, Virus-Induced Metabolism, and Other Cellular Functions

Plant viruses’ entry into vector cells induces a multitude of responses. Virus replication occurs within the cells of insect vectors of persistent-propagative viruses. Consequently, the presence of viruses in cells modulates several metabolic processes.

In whiteflies that acquired a semi-persistent virus, the Wnt and farnesyl pyrophosphate were upregulated in *B. tabaci* (MEAM1) that acquired ToCV from infected tomato plants [46]. Wnt transcription factors inhibited replication of human T-cell leukemia virus type 1 (HTLV-1), and farnesyl pyrophosphate interacted with HTLV-1 proteins [142,143]. In another whitefly cryptic species (*B. tabaci* (MED)) that acquired ToCV, eukaryotic translation initiation factor genes were identified among the upregulated DEGs [48]. Those genes were also reported to inhibit the replication of viruses such as influenza A by upregulating the expression level of interferon-induced transmembrane protein 3 [144]. The role of the replication-inhibiting genes indicated above is unclear in the case of semi-persistently transmitted foregut-borne criniviruses, as criniviruses do not replicate within whiteflies. In *B. tabaci* (MEAM1) that acquired another semi-persistent virus (CYSDV) from infected melon plants, 10 ATPases Associated with cellular Activities (AAA-ATPases) were identified among the downregulated genes [47]. AAA-ATPases are involved in functions such as cell-cycle regulation and vesicle-mediated protein transport [138,145].

The acquisition of a persistent-propagative virus, MMV, in the corn planthopper (*P. maidis*) resulted in the upregulation of RNA-dependent DNA polymerase (RdRp) genes in the vector, and RdRp is reported to facilitate virus infection and propagation [73]. In the case of another persistent-propagative virus, TSWV, Shrestha et al. found virus inhibitory protein endoplasmic reticulum-associated interferon-inducible (viperin) transcripts in the non-vector *F. tritici* but not in the vector *F. fusca* [114]. Although the role of viperin is not functionally validated in thrips, viperin is shown to suppress replication of influenza and rabies viruses as well as lentiviruses [146,147].

The viral activation of cell metabolism provides an increase in the pool of free nucleotides necessary for rapid viral genome replication, as well as increased amino acid production for rapid virion assembly [148]. In the vectors of persistent-propagative viruses, the differential regulation of metabolism-related genes was identified. Han and Rotenberg found that metabolism-associated DEGs, such as putative beta-glucosidase and acyl-CoA desaturase, were mostly downregulated in TSWV-infected *F. occidentalis* [64]. However, in another congeneric species, *F. fusca* infected with TSWV, carbohydrate metabolism genes were upregulated [47,65]. In *L. striatellus* infected with RSV, another persistent-propagative virus, glyoxylate and dicarboxylate metabolism genes were upregulated in both the alimentary canal and salivary glands [68,69]. In *G. nigrifrons* infected with the maize fine streak virus (MFSV), Cassone et al. found that genes associated with general metabolism were upregulated [49].

The upregulation of virus replication as well as nutrients’ metabolism gene transcripts in the case of persistent-propagative viruses falls in line with the accepted paradigm; however, the differential expression of such genes in the case of non-propagative semi-persistent viruses indicates an anomaly and remains to be investigated.

## 4. Immune Responses

**Figure 5:**
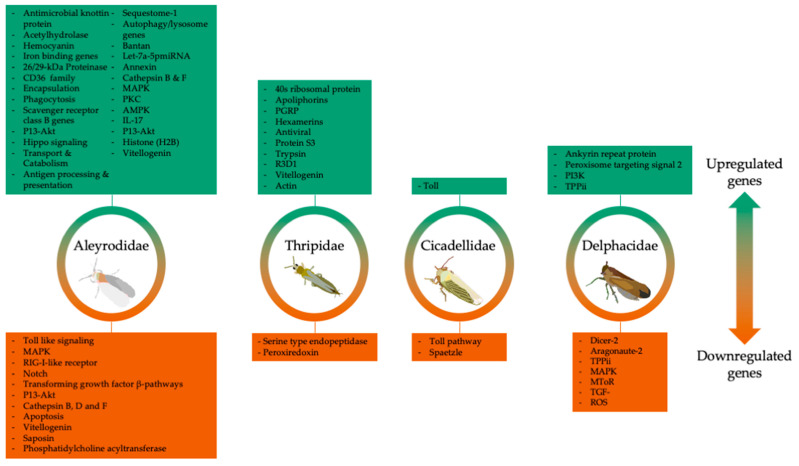
Major immune-related genes differentially expressed in four insect families. Direction of regulation was variable between insect families, with Aleyrodidae (whiteflies) showing the most identifiable changes across studies.

Upon the detection of the encapsulation proteins of plant viruses, a suite of vectors’ immune responses can be triggered. Below are the different immune genes and pathways that were differentially altered in vectors following the acquisition of plant viruses (Figure 5).

### 4.1. Inducible Humoral Response

Humoral response is mediated by antibodies, complement proteins, and antimicrobial peptides. These responses seem to be widespread, regardless of the mode of virus transmission.

In *B. tabaci* (MEAM1) that acquired a semi-persistent virus from CYSDV-infected melon plants, phosphatidylethanolamine-binding proteins (PEBP) genes were downregulated [47]. PEBPs play an important role in the innate immunity of insects [51,55]. An earlier study reported the downregulation of PEBP genes in *B. mori* strain resistant to BmNPV. Wang et al. speculated that the downregulation of PEBP could induce enhanced apoptosis, thereby repressing the ability of BmNPV to infect other cells [149]. In *B. tabaci* (MED) that acquired another semi-persistent virus, ToCV, hemocyanin transcripts were downregulated [48]. Hemocyanins are glycoproteins present in the hemolymph and have been associated with immune responses in insects such as fruit flies, mosquitoes, and psyllids [150,151,152]. Since semi-persistent viruses are limited to the vector’s foregut and do not cross into the hemolymph, the role of PEBPs and hemocyanins in whiteflies that acquire CYSDV and ToCV is rather unclear.

Antimicrobial knottin proteins, platelet-activating factor acetyl hydrolase, and a 26/29-kDa proteinase were other DEGs upregulated in *B. tabaci* (MEAM1) (whole bodies and midgut tissues) that acquired a persistent circulative virus from TYLCCNV-infected tobacco plants [51,55]. The activation of the knottin protein genes led Luan et al. to speculate that this was a strategy evolved by *B. tabaci* (MEAM1) to degrade virions [51]. Platelet-activating factor acetyl hydrolase gene was reported as a scavenger of oxidized phospholipids produced by pathogens, including viruses [52,153], while the 26/29-kDa proteinase was speculated to be associated with elimination of xenobiotic proteins in flesh fly, *Sarcophaga peregrina* (Robineau-Desvoidy) [50,154]. In whiteflies that acquired another persistent circulative virus from TYLCV-infected tomato plants, hemocyanins and iron binding genes were among the identified upregulated DEGs through transcriptomics [48,54]. Iron-binding proteins’ main role in insects is iron transport and immunity [155,156]. The upregulation of hemocyanins and iron binding genes in *B. tabaci* MEAM1 and MED, respectively, led researchers to speculate about their role in defense response to virus infection [48,54].

In thrips that acquired a persistent-propagative virus, TSWV, from infected plants, several defense and immune response genes, such as apolipophorins, hemocyanins, serine type endopeptidases, peroxiredoxins, peptidoglycan recognition proteins (PGRPs), lysozymes, and trypsin were identified among DEGs [60,63,64]. Schneweis et al. showed that TSWV infection in *F. occidentalis* increased the expression of phenoloxidase activity in melanogenesis [63]. Medeiros et al. found that 75% of the DEGs identified in TSWV-infected *F. occidentalis* adults were related to immune function [60]. In the Shrestha et al.’s study, different developmental stages in TSWV-infected *F. fusca* showed varying regulation patterns, such as the downregulation of genes associated with proteolysis (serine type endopeptidase) and antioxidant defense (peroxiredoxin) exclusively in the larval stage [65]. The few upregulated transcripts identified in the pupal stage were associated with PGRP immune gene homologs [114]. In another example, RSV acquisition in planthoppers caused an upregulation of PGRP and gram-negative bacteria-binding proteins’ genes in the salivary glands [69]. Furthermore, an increase in the expression of PGRP genes in *G. nigrifrons* that fed on MFSV-infected maize within 4 hr of feeding was observed [49]. By contrast, a noticeable decrease in the expression levels of three of the four PGRP genes in *G. nigrifrons* designated as transmitters that fed on a MFSV-infected maize was reported [74].

Vectors of persistently-transmitted viruses (non-propagative and propagative) shared only one gene: hemocyanin. As expected, the magnitude of the response was greater in the case of the persistent viruses versus non-persistent viruses, presumably triggered by the persistence of viruses within vectors and increased interactions with multiple tissues.

### 4.2. Signaling Responses

Signaling responses are initiated upon the detection of virions, and they range from the suppression of immune responses to tumorigenesis. Listed below are the various responses in different vectors upon detecting plant viruses.

In *B. tabaci* (MEAM1) that acquired ToCV, a semi-persistent virus, several genes involved in the phosphoinositide 3-kinase-protein kinase B (PI3K-Akt), Hippo signaling, transport and catabolism, and antigen processing pathways were identified [46]. The modulation of PI3-Akt in the influenza A and foot-and mouth disease virus and the Hippo pathway in Kaposi sarcoma-associated herpesvirus (KSHV) influenced virus penetration into host cells, the promotion of cell death, and tumorigenesis [157,158]. In the transport and catabolism category, different sets of lysosome DEGs at 24 hr and 72 hr post-acquisition led Kaur et al. to link the upregulation of these genes with virus uptake and downregulation with virus detachment/release post-acquisition [46]. Cathepsins are proteases that perform several roles, including apoptosis, autophagy, and innate immunity [159,160,161,162,163]. In *B. tabaci* (MED) that acquired ToCV, transcripts of cathepsins B and F were downregulated [48]. On the other hand, the annexin gene was among the identified upregulated genes in *B. tabaci* (MED) that acquired ToCV [48]. Annexin also was found to function in the apoptosis pathway [164].

In *B. tabaci* (MEAM1) that acquired a persistent circulative virus, TYLCCNV, from infected tomato plants, several genes involved in signaling pathways, such as toll-like signaling, mitogen-activated protein kinase (MAPK), retinoic acid-inducible gene I (RIG-I-like) receptor, Notch, and transforming growth factor beta were downregulated [51]. Signaling transduction pathways can trigger antiviral responses in insects [165,166]. The downregulation of signaling pathways was associated with the suppression of *B. tabaci* (MEAM1) immunity, thereby enabling the circulative movement of the virus in *B. tabaci* (MEAM1) [51,165,166]. The upregulation of DEGs in MAPK and protein kinase c (PKC) pathways facilitated immune system activation in whiteflies that acquired TYLCCNV [51]. Furthermore, the MAPK and PKC pathways were implicated in the membrane trafficking of adenovirus type 2 and influenza virus and the blockage of West Nile virus entry into mosquito cell line [167,168,169]. Most autophagy/lysosome genes were upregulated, and genes were reported to combat virus infection in general [170,171,172]. The upregulation of these genes in *B. tabaci* (MEAM 1) led Luan et al. to conclude that this was an immune response strategy evolved to offset the deleterious effects of the begomovirus [51]. In addition, several genes involved in the apoptosis pathway in viruliferous *B. tabaci* (MEAM1) were downregulated. Luan et al. associated the downregulation of the apoptosis pathway to TYLCCNV spread or movement within *B. tabaci* [51]. In another study by Luan et al., only three genes encoding lysosomal proteins, cathepsin D, phosphatidylcholine acyltransferase, and saposin, were downregulated in *B. tabaci* (MEAM1) that acquired TYLCCNV from infected tobacco plants, while the others, such as AP-1, cathepsin B, iduronate 2-sulfatase, protein tyrosine phosphatase, and vacuolar ATP synthase subunit S1, were upregulated [52].

Cathepsins B and F transcripts were downregulated in another whitefly cryptic species adults, *B. tabaci* (MED), that acquired TYLCV from infected tomato plants [48]. *B. tabaci* (MEAM1) that acquired another persistent circulative virus, TYLCV, from infected tomato plants included several DEGs involved in the 5′ adenosine monophosphate-activated protein kinase (AMPK), interleukin 17 (IL-17), and PI3K-Akt pathways [56]. Li et al. speculated that the downregulation of signaling pathways in TYLCV-infected *B. tabaci* (MEAM1) might aid in the persistence of the virus through the suppression of the vector’s immune system [56]. Further, DEGs associated MAPK and PKC pathways were upregulated in the midgut of *B. tabaci* (MEAM1) that acquired TYLCV [55].

In *F. occidentalis* that acquired a persistent-propagative virus (TSWV) from infected tomato plants, 40S ribosomal protein S3, which is involved in the regulation of apoptosis and the regulation of factors in the Toll and Imd pathways, was upregulated [61]. RSV, another persistent-propagative virus, infection in planthopper-repressed genes associated with MAPK, mTOR, and transforming growth factor beta (TGF-β) pathways [69]. In MMV-infected *G. nigrifrons*, innate immune genes, such as ankyrin repeat protein and peroxisomal targeting signal 2, were upregulated [73]. In *G. nigrifrons* infected with MFSV, genes associated with the Toll pathway were upregulated [49]. Chen et al. found that *G. nigrifrons* infected with MFSV reported a significant suppression of transcripts such as Toll and spaetzle [74].

Keeping in line with this trend, more signaling pathways were differentially affected following virus acquisition in the case of persistent viruses as opposed to semi-persistent viruses. The activation of a multitude of pathways, even in the case of non-propagative viruses, suggests robust evidence for an active immune system. The costs of the activation of these pathways in terms of their vectors’ fitness are not clear.

### 4.3. Cellular Responses

Following the acquisition of ToCV, the histone H2B gene was upregulated in *B. tabaci* (MED) that acquired ToCV [48]. This gene was reported to play a role in DNA repair inhibition [173].

Following the acquisition of a persistent virus (TYLCV) in *B. tabaci* (MEAM1), several DEGs associated with the CD36 family (scavenger receptor class B genes), encapsulation, and phagocytosis were upregulated in the midgut [55]. In *B. tabaci* (MED), scavenger receptor class B genes and sequestosome-1 were among the DEGs identified in viruliferous *B. tabaci* [48]. The scavenger class B receptor is a regulator of phagocytosis [55,174], and its upregulation in TYLCV-infected *B. tabaci* (MEAM1) was associated with resistance to the begomovirus [51,53]. The upregulation of sequestome-1, an autophagosome cargo protein in *B. tabaci* (MEAM1) that acquired TYLCV, was associated with resistance to the begomovirus [51,53].

Major components in insect immune defense mechanisms, pathogen-associated molecular patterns (PAMPs), were found to be involved in the initiation of phagocytotic gene activation [175]. Whitfield et al. found that the expression of autophagy-specific gene 3 (ATG 3), phosphoinositide 3-kinase (PI3K), and tripeptidyl peptidase ii (TPP ii) were elevated in MMV infected *P. maidis* [67]. Martin et al. found that autophagy-related genes, such as the peroxisomal targeting signal 2 receptor, were upregulated in MMV-infected *P. maidis* [73]. Evidence for phagocytosis was more commonly detected in the case of persistent viruses, as opposed to the semi-persistent viruses.

### 4.4. RNAi Responses

The sequence-specific suppression of gene expression by double-stranded RNA is another generic mechanism insect vectors deploy to defend cells against viruses. Wang et al. identified the upregulation of bantam and let-7a-5p microRNAs (miRNA) in *B. tabaci* (MEAM1) that acquired TYLCCNV from infected tomato plants [53]. The bantam miRNA was reported to simultaneously stimulate cell proliferation and prevent apoptosis in response to patterning cues in *Drosophila melanogaster* (Meigen) [176]. Wang et al. linked the enhanced expression level of bantam miRNA to its ability to arrest apoptotic response and help maintain homeostasis in the presence of the virus in *B. tabaci* [53].

The immune response regulation in *S. furcifera* showed that reactive oxygen species (ROS)-associated genes were suppressed by the presence of a persistent-propagative virus, SRBSDV [72]. A major component that was silenced was the Dicer-2 (DCR2) and Argonaute-2 (AGO2), which may have facilitated SRBSDV propagation in the midgut epithelium. There was a marked reduction of 60 to 70% in the abundance of RNAi-related genes, such as DCR2 and AGO2, in SRBSDV-infected *L. striatellus* and *S. furcifera* cells [71]. Similarly, Chen et al. found that *G. nigrifrons* infected with MFSV reported a significant suppression of transcripts such as DCR-2 and AGO-2 [73].

Comparisons of gene expression levels in non-viruliferous versus viruliferous *S. furcifera* showed high downregulation of functionally annotated genes associated with oxidative-reduction, response to oxidative stress, and translation [72]. Within the total number of 551 DEGs in RSV-infected *L. striatellus*, only four genes were predicted to be potential binding sites for the 70 virus-derived small interfering RNAs (vsiRNAs) [70]. Genes such as R3D1 associated with the RNA interference pathway (RNAi) were upregulated in TSWV-infected *F. fusca* [62]. The downregulation of these RNAi genes was observed in *D. melanogaster* and coincided with reduction in antivirus defense [165].

## 5. Vector Biological-Fitness-Related Genes

Vitellogenin-B (Vg-B), a gene associated with fecundity, lifespan, and other housekeeping roles, was the only reported downregulated DEG relating to biological fitness in *B. tabaci* (MEAM1) that acquired a semi-persistent virus, ToCV [46]. Reproductive vitellogenin (Vg) and development-related genes (juvenile hormone inducible protein genes) were differentially expressed in *B. tabaci* (MED) that fed on ToCV-infected tomato plants [48]. The upregulation of these genes in *B. tabaci* (MED) that acquired ToCV pointed to their role in shorter development time on ToCV-infected tomato plants than on non-infected tomato plants [177].

Wang et al. reported the upregulation of let-7a-5p microRNAs (miRNA) and linked it to perturbation of the cell cycle and negative effects on the longevity and fecundity of *B. tabaci* (MEAM1) that fed on TYLCCNV-infected tomato plants [53]. Vg genes were among the upregulated DEGs identified in viruliferous *B. tabaci* (MED) that acquired TYLCV, a persistent virus, from infected tomato plants [48]. Su et al. found an upregulation of two Vg genes in TYLCV-infected *B. tabaci* (MED) and attributed it to an increase in the fecundity of viruliferous *B. tabaci* (MED) [178]. Several genes associated with amino acid, carbohydrate, and lipid metabolism were differentially expressed in *B. tabaci* (MEAM1) that fed on TYLCV-infected tomato plants [56]. The downregulation of these genes was linked to deleterious effects in *B. tabaci*, such as reduced fecundity and longevity [179,180].

Shrestha et al. found that Vg and actin were both upregulated in *F. fusca* infected with a persistent-propagative virus, TSWV [65]. The majority of the contigs categorized as being involved in reproduction, embryo development, cell differentiation, and growth were upregulated in the viruliferous adults and pupae [65]. Cuticular proteins were among the downregulated transcripts not only in TSWV-infected *F. fusca*, but also in *S. furcifera* and *L. striatellus* that fed on SRBSDV-infected and RSV-infected rice plants, respectively [63,70,72]. Cuticular proteins are integral components of the membrane, and their downregulation implied a slowing-down of *T. palmi* development to facilitate virus acquisition [66]. Fifty-four transcripts associated with Vg were among the upregulated genes in CaCV-infected thrips [66]. Lee et al. found that Vg in viruliferous *L. striatellus* was upregulated, indicating that the virus could promote oogenesis to increase the frequency of transovarial transmission [68,181].

While there is substantial evidence of the upregulation of important reproductive hormones, such as vitellogenin, in vectors of persistent viruses, their upregulation in foregut-limiting viruses prompts us to reexamine the correlation. It is not clear how much of this upregulation is direct virus modulation, as opposed to indirect modulation via the host plant. Providing a short acquisition access on infected plants and gut clearing on non-infected plants could be a viable option to exclusively assess persistent virus-modulated effects, even though, in such scenarios, host effects cannot be completely excluded. Therefore, it becomes imperative to point out such caveats before studies attribute these fitness effects to direct virus modulation on their vectors.

## 6. Discussion

### 6.1. Vector Expression Profiles

Across a variety of transcriptomic studies considered for this review, 31 studies were selected, all of which met certain criteria for inclusion. These criteria limited the studies to those with transcript expression reported in insect vectors in relation to plant-transmitted viruses. Within all the studies considered in this review, more trends were observed in some DEGs than in others. Conversely, there were some DEGs that were regulated in the opposite direction when considering all the insect vectors.

A limited number of shared DEGs in each of the non-persistent, semi-persistent, and persistent non-propagative, and persistent-propagative categories highlighted in this review were recorded. This limited number of shared DEGs across several studies could have been driven by the duration of the interaction between the virus and vector. Under virus receptor gene families, several DEGs were identified in the different studies reviewed here; however, only the cuticular and transport-related genes were shared across a few transcriptome studies [46,54,55,56,65,70,72]. The orphan genes and glucose transporters associated with virus infection were the only identified DEGs that were shared across two transcriptome studies [46,48]. Among the biological-fitness-related genes, vitellogenin was the only identified DEG shared across four studies, which were focused on whiteflies infected with ToCV or TYLCV and thrips infected with TSWV [46,48,56,65]. Overall, the functional annotations of DEGs in the vectors of plant viruses were based on the gene functions associated with vectors of propagative animal viruses. Their role might be the same in the vectors of plant viruses, especially those that are non-propagative.

The observed similarities and differences in the direction of the expression of a given gene may have a relation to the evolutionary divergence times between the various insect vectors. Some genes may be more highly conserved than others and account for similarities in responses to infection. In some cases, the DEGS in non-persistently transmitted viruses were similar to those found in other modes of transmission. These differences may also be attributed to the limitations of the techniques used at the time of each study. Another factor to consider is the limitations of the respective databases and datasets available at the time of each study. As molecular techniques advance and more curated databases become available, the expression profiles of more vectors increase our understanding of the effects attributed to viral infection.

The DEG profile can be challenging to compare across a wide range of insect species and various methods that are specific to each study. To normalize the comparisons between each study, the main concepts regarding gene regulation were approached with a few key features in mind in this review. The genes described were those that were mainly associated with immune response, development, and reproduction. While the magnitude of expression is important, this was usually inconsistent across studies, which could in part be due to differing methods. However, genes that tended to be upregulated or downregulated were repeatedly mentioned across most of the studies examined in this review. Nevertheless, transcriptomic studies that point toward the overall picture of expression are as good as the baseline from which those studies are built. This baseline is the presence or absence of a well-curated database or genome.

### 6.2. Implications of the Common Genes/Pathways

A large amount of success has been witnessed in the potential uses of RNAi to disrupt the regulatory elements in insects [182]. In this review, several genes were discussed that were found to be involved in virus transmission that could be potential targets for RNAi. Insects that transmit disease-causing viruses cause significant amounts of damage every year across a wide range of important agricultural crops and their management is crucial. One such application involves the use of exogenous dsRNA to bind to regions of the virus genome and inhibit the production of binding proteins [183]. Techniques or formulations such as BioClay, which are layered double-hydroxide sheets used to coat plant leaves and exogenously deliver dsRNA to insect vectors, such as aphids, upon feeding are gaining traction [183]. Furthermore, exogenous applications are being explored for crop protection [183,184].

Genome-editing platforms, such as clustered, regularly interspaced, short palindromic repeats (CRISPR)-Cas9 may not be at the point at which developed technologies can be immediately or commercially applied in the field. However, techniques such as RNAi have been studied more, not only in laboratory settings but also in field situations [185]. While it will take time to understand CRISPR in the same sense, its high specificity and ability to edit large or small genomic regions of species of interest could provide a way to increase the effectiveness of RNAi [186,187,188]. Although RNAi technology looks to be promising, it also comes with the challenges of determining the most effective delivery method and screening for off-target effects [182,189,190]. As new genome editing tools are developed, the foundations of fully sequenced genomes and de novo assembled transcriptomes could be used to target specific genetic sites. More investigations into the transcriptomic profiles of insect vectors will help to develop practical applications for controlling vectors’ populations and/or managing devastating plant viruses in production systems. Future applications may result in genetically modified vectors that, by altering or eliminating binding proteins, could prevent the acquisition of viruses.

## Figures and Tables

**Table 2:** Summary of insect development stage, number of differentially expressed genes, and sequencing technology used in the reviewed studies.

Species	Developmental Stage Sampled	AAP: #Upregulated Genes	AAP: #Downregulated Genes	Sequencing Details	Reference
Non-persistent					
*Myzus persicae*	Adult	24 hr: 9732	24 hr: 10,818	Paired-end RNA-Seq-Illumina	[45]
Semi-persistent					
*Bemisia tabaci* (MEAM1)	Adult	24 hr: 447	24 hr: 542	Paired-end RNA-Seq-Illumina	[46]
	Adult	48 hr: 4	48 hr: 7		
	Adult	72 hr: 50	72 hr: 160		
*Bemisia tabaci* (MEAM1)	Adult	24 hr: 0	24 hr: 3	Paired-end RNA-Seq-Illumina	[47]
	Adult	72 hr: 82	72 hr: 139		
	Adult	7 d: 49	7 d: 2		
*Bemisia tabaci* (MED)	Adult	24 hr: 88	24 hr: 133	Paired-end RNA-Seq-Illumina	[48]
*Graminella nigrifrons*	Fifth-instar nymph	4 hr: 240	4 hr: 49	Paired-end RNA-Seq-Illumina	[49]
		7 d: 129	7 d: 407		
Persistent non-propagative					
*Bemisia tabaci* (MEAM1)	Adult	30 d: 124	30 d: 122	Expressed sequence tagsSanger sequencing	[50]
*Bemisia tabaci* (MEAM1)	Adult	24 hr: 840	24 hr: 766	Single-end RNA-SeqIllumina	[51]
*Bemisia tabaci* (MEAM1)	Adult	25 d: 140	25 d: 317	Single-end RNA-SeqIllumina	[52]
*Bemisia tabaci* (MEAM1)	Adult	24 hr: 15	24 hr: 9	Solexa sequencing	[53]
*Bemisia tabaci* (MEAM1)	Adult	24 hr: 20	24 hr: 18	Paired-end RNA-SeqIllumina	[54]
		48 hr: 0	48 hr: 7		
		72 hr: 16	72 hr: 21		
*Bemisia tabaci* (MEAM1)	Adult	24 hr: 4014	24 hr: 1193	Paired-end RNA-SeqIllumina	[55]
*Bemisia tabaci* (MED)	Adult	24 hr: 43	24 hr: 35	Paired-end RNA-SeqIllumina	[48]
*Bemisia tabaci* (MEAM1)	Adult	2 hr: 513	2 hr: 242	Paired-end RNA-SeqBGISEQ-500	[56]
		6 hr: 299	6 hr: 288		
		24 hr: 388	24 hr: 752		
		48 hr: 391	48 hr: 956		
*Schizaphis graminum*	Adult (parental)	5 d: 68	NA	Two-dimensional difference gel electrophoresis	[57]
	Adult (F2)	5 d: 14	NA		
*Acyrthosiphon pisum*	Adult	2–6 d: 23	2–6 d: 105	RT-PCR and Microarrays	[58]
*Sitobion avenae*	Adult	Reared first-instar: 296	Reared first-instar: 296	Paired-end RNA-SeqIllumina	[59]
Persistent-propagative					
*Frankliniella occidentalis*	First-instar larva	Combined 12 and 96 hr: 51	NA	PCR and Microarrays	[60]
*Frankliniella occidentalis*	Adult	3 hr: 10	3 hr: 16	Pyrosequencing454	[61]
*Frankliniella occidentalis*	First- and second-instar larva, pre-pupa and pupa, and adult	48 hr: 661	48 hr: 793	Paired-end RNA-SeqIllumina	[62]
*Frankliniella fusca*	First- and second-instar larva	3 hr: 219	3 hr: 176	Paired-end RNA-SeqIllumina	[65]
	Pre-pupa and pupa	3 hr: 204	3 hr: 54		
	Adult	3 hr: 478	3 hr: 84		
*Frankliniella occidentalis*	First- and second-instar larva	3 hr: 17	3 hr: 161	Single-end RNA-SeqIllumina	[63]
	Pre-pupa and pupa	3 hr: 92	3 hr: 89		
	Adult	3 hr: 59	3 hr: 68		
*Frankliniella occidentalis*	First- and second-instar larva	1 d: 60	1 d: 101	Paired-end RNA-SeqIllumina	[64]
*Thrips palmi*	Adult	24 hr: 708	24 hr: 681	Paired-end RNA-SeqIllumina	[66]
*Nilaparvata lugens*	Fourth- and fifth-instar nymph	2 wk: 2 *	2 wk: 2 *	RT-qPCR	[67]
*Laodelphax striatellus*	Adult	Field collected: 453	Field collected: 428	Pyrosequencing454	[68]
*Laodelphax striatellus*	Second-instar nymph	1 d: 4 *	NA	Solexa sequencing	[71]
*Laodelphax striatellus*	Adult	8 d: 603	8 d: 1081	Single-end RNA-SeqIllumina	[69]
	Adult	8 d: 146	8 d: 81		
*Sogatella furcifera*	Second-instar nymph	2 d: 278	2 d: 406	Paired-end RNA-SeqIllumina	[72]
*Peregrinus maidis*	Adult	7 d: 76	7 d: 68	Single-end RNA-SeqIllumina	[73]
*Laodelphax striatellus*	Fourth-instar nymph	Field collected: 286	Field collected: 265	Paired-end RNA-SeqIllumina	[70]
*Graminella nigrifrons*	Adult	3 wk: 3 *	3 wk: 4 *	Paired-end RNA-SeqIllumina	[74]
*Graminella nigrifrons*	Fifth-instar nymph	4 hr: 636	4 hr: 121	Paired-end RNA-SeqIllumina	[49]
*Graminella nigrifrons*	Adult	21 d: 7 *	21 d: 3 *	RT-qPCR	[75]

* Limited expression reported. Abbreviations: AAP– acquisition access period, hr—hours, d—days, and wk—weeks.

## Supplementary Materials

The following supporting information can be downloaded at: https://www.mdpi.com/article/10.3390/cells11040693/s1. Figure S1: Deposition of gene annotation files uploaded to the National Center for Biotechnology Information (NCBI) gene database over time. Figure S2: Relative frequency of studies using RNA-Seq data derived from various tissue types. References [191,192,193,194,195,196,197] are cited in the supplementary materials.
