# Supplementary material for: A Review on Transcriptional Responses of Interactions between Insect Vectors and Plant Viruses
Source: Cells. 2022 Feb 16;11(4):693. doi: 10.3390/cells11040693 (PMC8870222; doi:10.3390/cells11040693)
Supplement: Supplementary file 1 [file cells-11-00693-s001.zip › cells-1541625-supplementary.pdf]

Supplementary file

Review

A review on transcriptional responses of interactions between insect vectors and plant viruses

Michael A. Catto [1,†], Habibu Mugerwa [2,†], Brendon K. Myers [3], Sudeep Pandey [2], Bhabesh Dutta [3], and Rajagopalbabu Srinivasan [2,*]

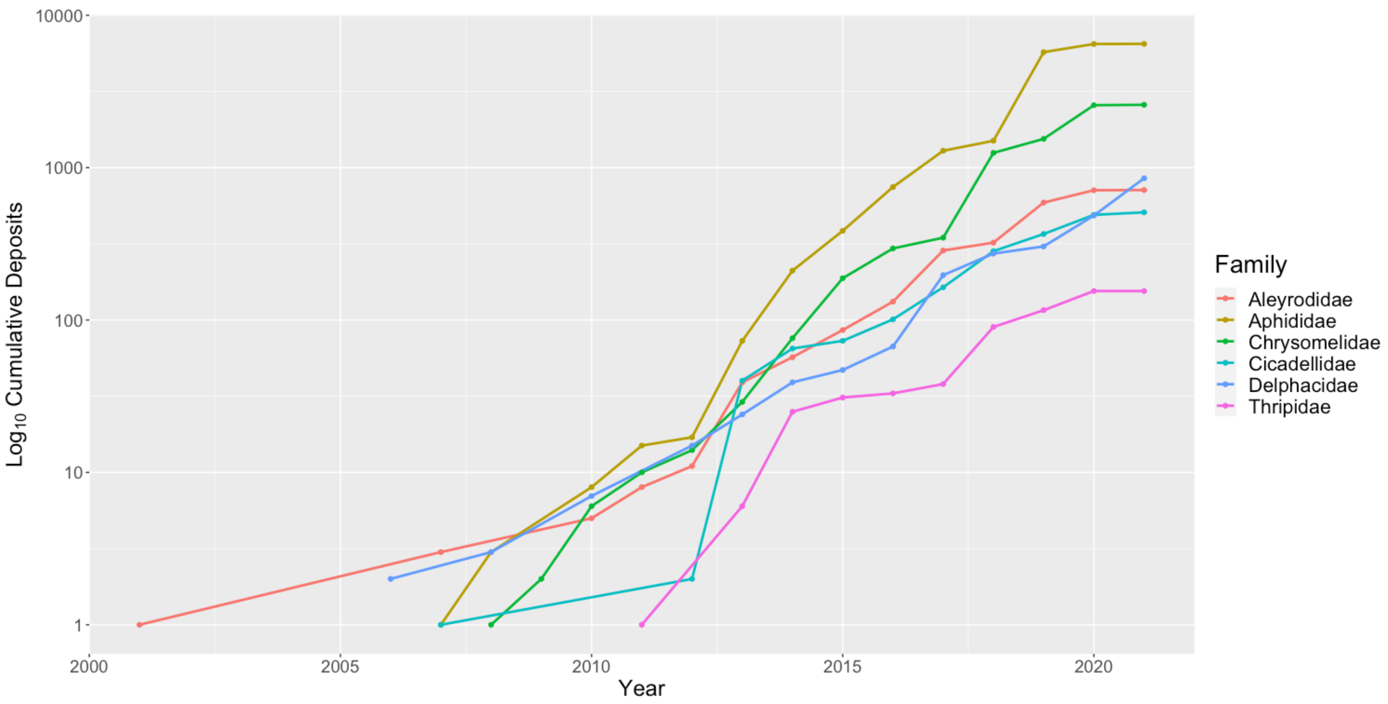

Figure S1. Deposition of gene annotation files uploaded to the National Center for Biotechnology Information (NCBI) gene database over time. Genes include mRNA from six families that are known to transmit viruses in non-persistent, semi-persistent, and persistent (non-propagative and propagative) modes. These data are representative of insects that may be viruliferous or non-viruliferous.

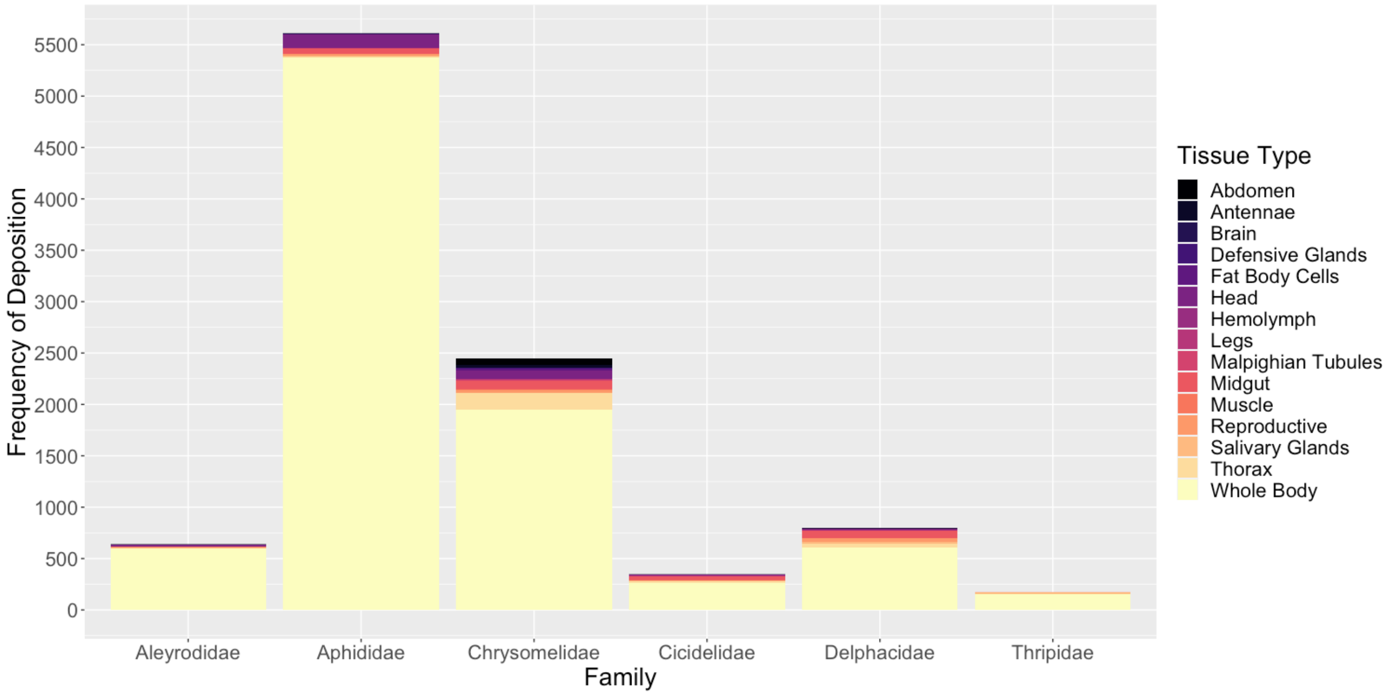

Figure S2. Relative frequency of studies using RNA-Seq data derived from various tissue types. These data were gathered from the NCBI Transcriptome Shotgun Assembly Database from published studies (n = ~10,000 samples). Most of the sequence data were obtained from the whole-body insect samples analyzed, whereas fewer studies have focused on specific tissue types. These data are representative of insects that may be viruliferous or non-viruliferous.

References and links to tools for a generic differential expression analysis pipeline outlined in **Figure 4**.

Drawio - https://github.com/jgraph/drawio

Illumina - https://www.illumina.com/

PacBio - https://www.pacb.com/

R - https://www.r-project.org/

FastQC - https://www.bioinformatics.babraham.ac.uk/projects/fastqc/

Trimmomatic - Bolger, A. M.; Lohse, M.; Usadel, B. Trimmomatic: A flexible trimmer for Illumina Sequence Data. *Bioinformatics* **2014**, *15*, 2114-2120, doi:10.1093/bioinformatics/btu170

Pilon - Walker, B.J.; Abeel, T.; Shea, T.; Priest, M.; Abouelliel, A.; Sakthikumar, S.; Cuomo, C.A.; Zeng, Q.; Wortman, J.; Young, S.K.; Earl, A.M. Pilon: An Integrated Tool for Comprehensive Microbial Variant Detection and Genome Assembly Improvement. *PLOS ONE* **2014**, *9*, e112963, doi:10.1371/journal.pone.0112963

Trinity - Grabherr, M.G.; Haas, B.J.; Yassour, M.; Levin, J.Z.; Thompson, D.A.; Amit, I.; Adiconis, X.; Fan, L.; Raychowdhury, R.; Zeng, Q.; Chen, Z.; Mauceli, E.; Hacohen, N.; Gnirke, A.; Rhind, N.; di Palma, F.; Birren, B.W.; Nusbaum, C.; Lindblad-Toh, K.; Friedman, N.; Regev, A. Full-length transcriptome assembly from RNA-Seq data without a reference genome. *Nature biotechnology* **2011**, *29*, 644–652. doi: 10.1038/nbt.1883

BUSCO - Simão, F.A.; Waterhouse, R.M.; Ioannidis, P.; Kriventseva, E.V.; Zdobnov, E.M. BUSCO: assessing genome assembly and annotation completeness with single-copy orthologs. *Bioinformatics* **2015**, *19*, 3210-3212. doi:10.1093/bioinformatics/btv351

DESeq2 - Love, M.I.; Huber, W.; Anders, S. Moderated estimation of fold change and dispersion for RNA-seq data with DESeq2. *Genome Biol* **2014**, *15*, 550, doi:https://doi.org/10.1186/s13059-014-0550-8

EdgeR - Robinson, M. D.; McCarthy, D. J.; Smyth, G. K. edgeR: a Bioconductor package for differential expression analysis of digital gene expression data. *Bioinformatics* **2010**, *1*, 139–140. doi:10.1093/bioinformatics/btp616
Blat2GO - Conesa, A.; Götz, S. Blast2GO: A comprehensive suite for functional analysis in plant genomics. *International journal of plant genomics* **2008**, 619832. doi:10.1155/2008/619832
